# Antimicrobial activity of dalbavancin against Gram-positive bacteria isolated from patients hospitalized with bloodstream infection in United States and European medical centers (2018–2020)

**DOI:** 10.1007/s10096-022-04437-0

**Published:** 2022-03-30

**Authors:** Helio S. Sader, Mariana Castanheira, Michael D. Huband, Dee Shortridge, Cecilia G. Carvalhaes, Rodrigo M. Mendes

**Affiliations:** JMI Laboratories, 345 Beaver Kreek Centre, Suite A, North Liberty, IA 52317 USA

**Keywords:** Dalbavancin, Lipoglycopeptide, *Staphylococcus aureus*, MRSA, Bacteraemia

## Abstract

Dalbavancin and comparators were susceptibility tested against 8643 Gram-positive bacteria from 74 hospitals located in Europe and the United States by broth microdilution method. The most common organisms were *Staphylococcus aureus* (45.2%), *Enterococcus faecalis* (12.2%), and *Staphylococcus epidermidis* (8.9%), but rank order varied markedly by geographic region. Dalbavancin demonstrated potent activity and broad spectrum, with MIC_90_ values of 0.03 mg/L for *Staphylococcus aureus*, β-haemolytic streptococci, and viridans group streptococci; 0.06 mg/L for *Enterococcus faecalis* and *Staphylococcus epidermidis*; and 0.12 mg/L for vancomycin-susceptible *Enterococcus faecium*. All organisms, except vancomycin-resistant enterococci and 1 *Staphylococcus haemolyticus* isolate, were inhibited at ≤ 0.25 mg/L of dalbavancin.

## Introduction

Bloodstream infection (BSI) includes a wide variety of syndromes caused by a range of pathogens; accordingly, BSI produces significant patient morbidity and mortality worldwide [1]. Changing pathogen distribution, antimicrobial resistance rates, and demographics may affect the epidemiology of BSI. Thus, it is important to continuously monitor trends in the pathogen frequency and antimicrobial resistance patterns of organisms causing BSI globally [2, 3]. Examining microbiological trends can help when planning diagnostic approaches, treatment strategies, and prevention programs.

The International Dalbavancin Evaluation of Activity (IDEA) Program monitors the in vitro activity of dalbavancin and comparators against Gram-positive bacteria causing BSI and other infections in Europe (EU) and the United States (US). Strengths of this surveillance program include the broad geographic distribution of medical centers submitting clinical isolates and the use of reference identification and antimicrobial susceptibility testing methods at a central laboratory [4].

Dalbavancin belongs to the lipoglycopeptide class of antimicrobial agents that act by interrupting bacterial cell wall synthesis resulting in bacterial death [5]. Dalbavancin allows for convenient parenteral administration to treat acute bacterial skin and skin structure infections, either through a single dose of 1500 mg or one dose of 1000 mg followed by another dose of 500 mg a week later [6, 7]. Dalbavancin was approved in the US (2014) and EU (2015) to treat adults with acute bacterial skin and skin structure infection (ABSSSI) caused by *Staphylococcus aureus*, including methicillin-resistant (MRSA) isolates, *Streptococcus pyogenes*, *Streptococcus agalactiae*, *Streptococcus dysgalactiae*, *Streptococcus anginosus* group, and vancomycin-susceptible *Enterococcus faecalis*. Dalbavancin is not licensed to treat patients with BSI, but it could be an important option to treat infections due to highly resistant Gram-positive cocci [8, 9]. It is also important to note that ABSSSI can be secondarily complicated by bacteremia or it can be the result of skin/subcutaneous tissue seeding during bacteremia from a distant focus. Furthermore, catheter-related infections may commonly present as both ABSSSI and BSI due to the same organism. In this investigation, we evaluated dalbavancin in vitro activity and potency when tested against a large collection of Gram-positive bacteria collected from patients with BSI in US and European medical centers.

## Materials and methods

### Organism collection

A total of 8643 organisms were consecutively collected (1/patient) from 74 medical centers located in western Europe (W-EU; *n* = 3330; 28 centers from 10 countries: Belgium, France, Germany, Ireland, Italy, Portugal, Spain, Sweden, Switzerland, and the UK), eastern Europe (E-EU; *n* = 769; 13 centers from 10 countries: Belarus, Czech Republic, Greece, Hungary, Israel, Poland, Romania, Russia, Slovenia, and Turkey), and the US (*n* = 4544; 33 centers). Isolates determined to be clinically significant based on local guidelines were submitted to a central monitoring laboratory (JMI Laboratories, North Liberty, IA, USA) [2]. Species identification was initially performed by the participating laboratories then confirmed at JMI Laboratories by standard algorithms and/or MALDI-TOF.

### Antimicrobial susceptibility testing

Isolates were susceptibility tested by broth microdilution following guidelines in the CLSI M07 document [10] with reference 96-well panels manufactured by JMI Laboratories. All isolates were tested at JMI Laboratories. Polysorbate-80 at a final concentration of 0.002% was added to the medium to test dalbavancin. Isolates with elevated dalbavancin MIC values (> 0.25 mg/L) were retested to confirm the dalbavancin MIC results. Quality assurance was performed by concurrently testing the following CLSI-recommended quality control (QC) reference strains: *S. aureus* ATCC 29213, *E. faecalis* ATCC 29212, and *S. pneumoniae* ATCC 49619. All QC results were within published acceptable ranges. Dalbavancin breakpoints approved by the US FDA (≤ 0.25 mg/L) [6], CLSI (≤ 0.25 mg/L) [11], and EUCAST (≤ 0.125 mg/L) [12] were applied when appropriate. US FDA, CLSI, and EUCAST breakpoint criteria were used for the comparator agents.

## Results

**Fig. 1:**
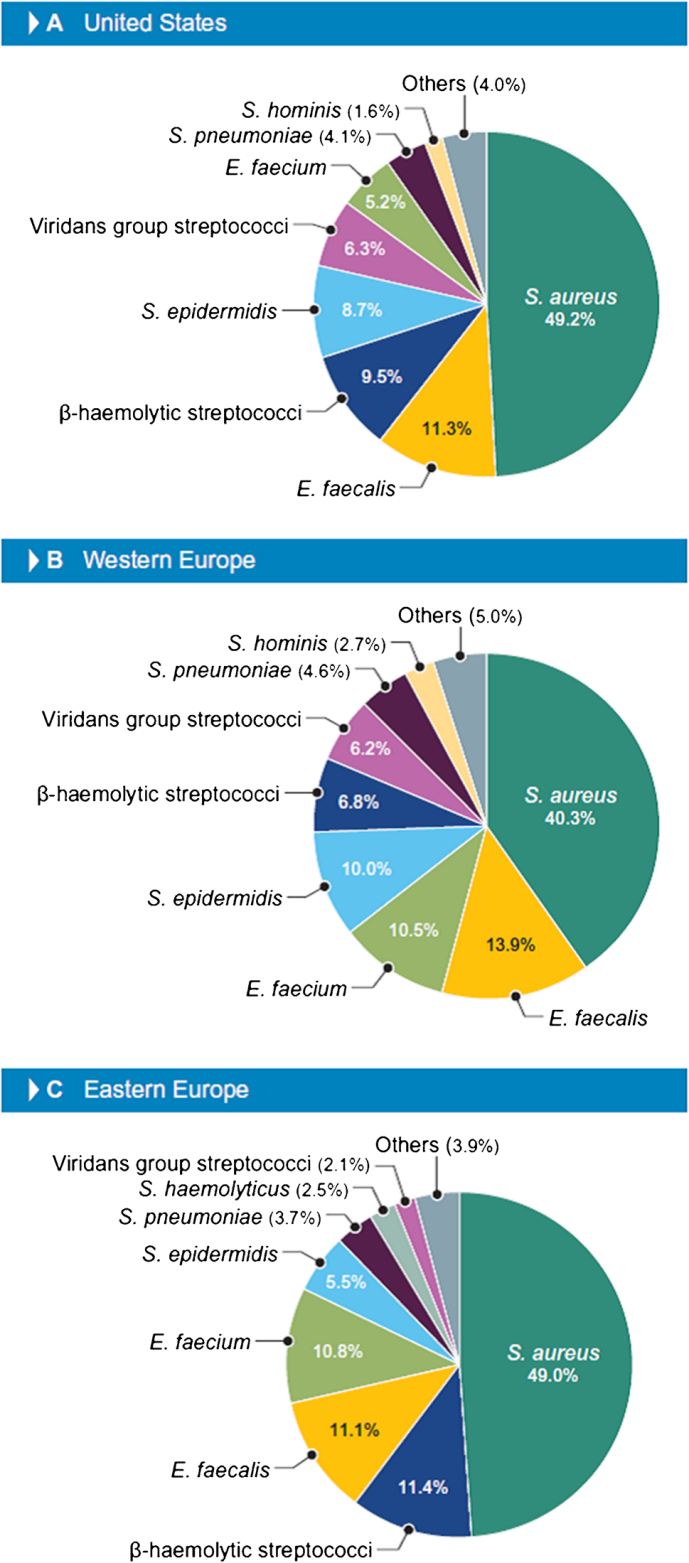
Frequency of Gram-positive bacteria isolated from patients hospitalized with bacteremia in the United States (US), western Europe (W-EU), and eastern Europe (E-EU) in 2018–2020

Overall, the most common Gram-positive organisms were *S. aureus*, *E. faecalis*, *S. epidermidis*, β-hemolytic streptococci (BHS), and *E. faecium*, but rank order varied markedly by geographic region (Fig. 1). *S. aureus* ranked first in all 3 regions, with frequencies varying from 49.2% in the US to 40.3% in W-EU. The second most common organism was *E. faecalis* in the US and W-EU and *S. pneumoniae* in E-EU. The third most frequently isolated Gram-positive organism was BHS in the US and E-EU and *E. faecium* in W-EU (Fig. 1).

Dalbavancin was highly active against methicillin-susceptible *S. aureus* (MSSA) and MRSA, with an MIC_90_ of 0.03 mg/L in all 3 regions and 100.0% susceptibility overall per US FDA, CLSI, and EUCAST criteria (Tables 1 and 2). Based on MIC_50/90_ values, dalbavancin (MIC_50/90_, 0.03/0.03 mg/L) was 8- to 16-fold more active than daptomycin (MIC_50/90_, 0.25/0.5 mg/L) and 32-fold more active than vancomycin (MIC_50/90_, 1/1 mg/L) against *S. aureus* (Table 2). MRSA rates were higher in the US (41.3%) than W-EU (21.5%) or E-EU (27.3%). *S. aureus* susceptibility to ceftaroline ranged from 96.6% (US) to 95.4% (W-EU), whereas *S. aureus* susceptibility to clindamycin and levofloxacin (US FDA and CLSI criteria) was lower in the US (85.5% and 67.6%, respectively) than W-EU (96.2% and 79.4%, respectively) and E-EU (89.1% and 85.8%, respectively; Table 2).

**Table 1:** Antimicrobial activity of dalbavancin tested against the most common organisms and organism groups

Organism (no. of isolates)	No. and cumulative % of isolates inhibited at dalbavancin MIC (mg/L) of:													
	≤ 0.004	0.008	0.015	0.03	0.06	0.12	0.25	0.5	1	2	> 2	MIC_50_	MIC_90_	
*S. aureus* (3908)	4 0.1	31 0.9	970 25.7	2,840 98.4	62 > 99.9	1 100.0						0.03	0.03	
MSSA (2607)	3 0.1	25 1.1	661 26.4	1882 98.6	35 > 99.9	1 100.0						0.03	0.03	
MRSA (1301)	1 0.1	6 0.5	309 24.3	958 97.9	27 100.0							0.03	0.03	
*E. faecalis* (1053)			159 15.1	752 86.5	117 97.6	6 98.2	1 98.3	0 98.3	0 98.3	1 98.4	17 100.0	0.03	0.06	
VAN-S (≤ 4 mg/L) (1030)			159 15.4	752 88.4	112 99.3	6 99.9	1 100.0					0.03	0.06	
*S. epidermidis* (765)	3 0.4	17 2.6	201 28.9	436 85.9	90 97.6	17 99.9	1 100.0					0.03	0.06	
β-hemolytic streptococci (735)	130 17.7	213 46.7	307 88.4	66 97.4	17 99.7	2 100.0						0.015	0.03	
*E. faecium* (659)			71 10.8	155 34.3	138 55.2	52 63.1	15 65.4	6 66.3	3 66.8	11 68.4	208 100.0	0.06	> 2	
VAN-S (≤ 4 mg/L) (397)			68 17.1	143 53.1	133 86.6	47 98.5	6 100.0					0.03	0.12	
Viridans group streptococci (508)	116 22.8	126 47.6	140 75.2	100 94.9	20 98.8	6 100.0						0.015	0.03	
*S. pneumoniae* (461)	12 2.6	245 55.7	187 96.3	16 99.8	1 100.0							0.008	0.015	
*S. hominis* (175)	1 0.6	4 2.9	52 32.6	95 86.9	19 97.7	4 100.0						0.03	0.06	
*S. haemolyticus* (104)			3 2.9	16 18.3	51 67.3	29 95.2	4 99.0	1 100.0				0.06	0.12	

Abbreviations: *VAN-S*, vancomycin-susceptible

**Table 2:** Antimicrobial activity of dalbavancin and comparator agents against the most common Gram-positive cocci isolated from patients with BSI in the United States (US), western Europe (W-EU), and eastern Europe (E-EU)

Organism/antimicrobial (no. tested)	MIC_50_^a^	MIC_90_^a^	% Susceptible per US FDA and CLSI (no. tested)		
			US	W-EU	E-EU
*S. aureus* (3908)			(2,235)	(1,343)	(330)
Dalbavancin	0.03	0.03	100.0	100.0	100.0
Daptomycin	0.25	0.5	> 99.9	100.0	100.0
Vancomycin	1	1	100.0	100.0	100.0
Teicoplanin	0.5	0.5	100.0	100.0	100.0
Linezolid	1	2	100.0	100.0	100.0
Oxacillin	0.5	> 2	58.7	78.5	72.7
Ceftaroline	0.25	1	96.6	95.4	96.4
Clindamycin	0.06	> 2	85.5	96.2	89.1
Levofloxacin	0.25	> 4	67.6	79.4	85.8
Tetracycline	≤ 0.5	≤ 0.5	95.1	96.0	83.6
TMP-SMX^b^	≤ 0.5	≤ 0.5	97.7	99.8	99.7
*E. faecalis* (1053)			(515)	(463)	(75)
Dalbavancin	0.03	0.06	97.9 ^c^	98.7 ^c^	98.7 ^c^
Daptomycin	1	1	99.2	99.6	100.0
Vancomycin	1	2	97.5	98.3	97.3
Teicoplanin	0.5	0.5	97.9	98.7	98.7
Linezolid	1	2	99.8	99.8	97.3
Ampicillin	1	1	100.0	100.0	100.0
Levofloxacin	1	> 4	78.8	73.4	70.7
*S. epidermidis* (765)			(396)	(332)	(37)
Dalbavancin	0.03	0.06	[100.0]^d^	[100.0]^d^	[100.0]^d^
Daptomycin	0.25	0.5	100.0	100.0	100.0
Vancomycin	2	2	100.0	100.0	100.0
Teicoplanin	2	8	99.2	99.4	97.3
Linezolid	1	1	93.9	96.4	94.6
Oxacillin	> 2	> 2	26.8	33.1	13.5
Clindamycin	0.06	> 2	52.5	66.6	70.3
Levofloxacin	4	> 4	40.9	44.6	24.3
Tetracycline	1	> 8	80.8	85.2	73.0
TMP-SMX^b^	1	8	54.3	58.4	73.0
β-hemolytic streptococci (735)			(430)	(228)	(77)
Dalbavancin	0.015	0.03	100.0^e^	100.0^e^	100.0^e^
Daptomycin	≤ 0.06	0.25	100.0	100.0	100.0
Vancomycin	0.5	0.5	100.0	100.0	100.0
Linezolid	1	2	100.0	100.0	100.0
Ceftriaxone	0.03	0.06	100.0	100.0	100.0
Ceftaroline	≤ 0.008	0.015	100.0	100.0	100.0
Penicillin	0.015	0.06	100.0	100.0	100.0
Clindamycin	≤ 0.25	> 2	79.8	87.7	85.7
Levofloxacin	0.5	1	98.1	97.4	98.7
Tetracycline	> 4	> 4	41.7	52.2	55.8
*E. faecium* (659)			(238)	(348)	(73)
Dalbavancin	0.06	> 2	[38.7]^c^	[81.9]^c^	[74.0]^c^
Daptomycin	1	2	[96.2]^f^	[100.0]^f^	[100.0]^f^
Vancomycin	0.5	> 16	36.6	76.1	61.6
Teicoplanin	1	> 16	39.9	82.2	67.1
Linezolid	1	2	99.2	99.7	100.0
Ampicillin	> 16	> 16	18.5	12.6	2.7
Levofloxacin	> 4	> 4	14.7	10.1	2.7

^a^MIC_50_ and MIC_90_ values for the US, W-EU, and E-EU collection combined

^b^Trimethoprim-sulfamethoxazole

^c^These breakpoints have been applied to all *E. faecalis* and *E. faecium* but are only approved for vancomycin-susceptible *E. faecalis*

^d^The percentage inhibited at ≤ 0.25 mg/L, the susceptible breakpoint for *S. aureus* published by US FDA and CLSI

^e^These breakpoints have been applied to all *Streptococcus* spp. other than *S. pneumoniae*, but are only approved for *S. pyogenes*, *S. agalactiae*, and *S. dysgalactiae* group

^f^The value in the brackets indicates percentage susceptible dose-dependent (SDD)

Vancomycin susceptibility varied from 97.3% (E-EU) to 98.3% (W-EU) among *E. faecalis* (97.5% in US; Table 2), and dalbavancin was highly active against vancomycin-susceptible *E. faecalis* (MIC_50/90_, 0.03/0.06 mg/L; 100.0% susceptible [S] per US FDA and CLSI [98.5% inhibited at ≤ 0.12 mg/L]; Table 1). Dalbavancin coverage against *E. faecalis* per US FDA and CLSI criteria (97.9–98.7%S) was identical to teicoplanin (97.9–98.7%S) and comparable to daptomycin (99.2–100.0%S), vancomycin (97.3–98.3%S), and linezolid (97.3–99.8%S); however, based on MIC_50_ values, dalbavancin was 16- to 32-fold more potent than those compounds (Table 2). All *E. faecalis* isolates were ampicillin susceptible (MIC_50/90_, 1/1 mg/L; Table 2).

*S. epidermidis* was the third most common organism overall but ranked fourth in the US and W-EU and sixth in E-EU (Fig. 1). Oxacillin resistance rates among *S. epidermidis* were 66.9% in W-EU, 73.2% in US, and 86.5% in E-EU, and all isolates were inhibited at ≤ 0.25 mg/L of dalbavancin (MIC_50/90_, 0.03/0.06 mg/L; 99.9% inhibited at ≤ 0.12 mg/L; Tables 1 and 2). Daptomycin (MIC_50/90_, 0.25/0.5 mg/L) and vancomycin (MIC_50/90_, 2/2 mg/L) were active against all *S. epidermidis*, whereas susceptibility to teicoplanin (US FDA and CLSI criteria) ranged from 97.3% (E-EU) to 99.2% (US) and 99.4% (W-EU) and susceptibility to linezolid ranged from 93.9% (US) to 96.4% (W-EU; Table 2).

BHS exhibited low dalbavancin MIC values (MIC_50/90_, 0.015/0.03 mg/L) and high susceptibility rates for most comparator agents tested (Tables 1 and 2). *E. faecium* ranked third in W-EU, fifth in E-EU, and sixth in the US, and showed vancomycin susceptibility rates of 76.1% in W-EU, 61.6% in E-EU, and only 36.6% in the US (Table 2). Dalbavancin inhibited 100.0% of vancomycin-susceptible *E. faecium* at ≤ 0.25 mg/L (98.5% inhibited at ≤ 0.12 mg/L) but exhibited very limited activity against vancomycin-resistant *E. faecium* (Table 1). Linezolid was the most active compound tested against *E. faecium* (MIC_50/90_, 1/2 mg/L; 99.5%S per US FDA and CLSI and 99.8%S per EUCAST; Table 2).

## Discussion

Dalbavancin is a long-acting lipoglycopeptide characterized by a long elimination half-life coupled with excellent in vitro activity against multidrug-resistant Gram-positives [5, 7]. Although dalbavancin has not been evaluated in clinical trials for BSI and it is currently approved only for the treatment of ABSSSI, dalbavancin has shown clinical efficacy and good tolerability for various infections. Some observational studies and real-world clinical experiences suggest the efficacy of dalbavancin for infections needing long-term treatment courses, including osteomyelitis, prosthetic joint infection, and endocarditis. In these studies, dalbavancin was used as either first-line agent or, more commonly, as consolidation to complete the treatment course and allow for an early discharge [9, 13, 14].

Data from the dalbavancin clinical trials, where all patients had blood cultures obtained at baseline, indicated that a total of 40 ABSSSI patients who received dalbavancin had bacteremia at baseline caused by one or more of the following organisms: 26 *S. aureus* (21 MSSA and 5 MRSA), 6 *S. agalactiae*, 7 *S. pyogenes*, 2 *S. anginosus* group, and 1 *E. faecalis*. Thirty-four of 40 (85.0%) patients who received dalbavancin showed favorable clinical responses at 48 to 72 h and 32/40 (80.0%) were clinical successes at days 26 to 30 [6, 15]. Moreover, the efficacy and safety of dalbavancin for the treatment of BSI and cardiovascular infections have been evaluated in many observational studies and case reports [9, 14, 16–20].

Gatti et al. recently summarized the results of 144 patients affected by BSI or vascular infection that were treated with dalbavancin. Different dalbavancin dosage treatment durations were administered. Clinical success was obtained in 81.3% of cases and relapse was reported in 3.5% of cases [9]. In a case of prosthetic graft infection due to *E. faecium*, dalbavancin was successfully administered as a long-term suppressive therapy for a total of 62 weeks [13]. In the DALBACEN cohort study, 49 patients affected by BSI that received at least one dose of dalbavancin were assessed. Dalbavancin was administered as a single dose of 1000–1500 mg, or 1000 mg followed by 500 mg at day 8. Clinical success was documented in 100.0% of patients at 90 days (including two cases of BSI caused by *E. faecium*), with no case of relapse or resistance development [18].

In the present investigation, dalbavancin demonstrated potent in vitro and broad-spectrum activity against Gram-positive organisms isolated from patients with BSI in European and US medical centers, with MIC_90_ values of 0.03 mg/L for *S. aureus*, BHS, and VGS; 0.06 mg/L for *E. faecalis* and *S. epidermidis*; and 0.12 mg/L for vancomycin-susceptible *E. faecium*. All organisms, except vancomycin-resistant enterococci and 1 *S. haemolyticus* isolate, were inhibited at the dalbavancin-susceptible breakpoint of ≤ 0.25 mg/L (US FDA and CLSI criteria). Additionally, dalbavancin MIC values were 8- to 16-fold lower than those of daptomycin and 32-fold lower than those of vancomycin when tested against *S. aureus.* These results are consistent with in vitro surveillance studies reported since 2002 and cited in several recent reviews. Additionally, these results indicate that resistance to other antimicrobial classes, with the exception of the VanA vancomycin-resistance phenotype, does not adversely affect dalbavancin activity [4, 8, 21, 22]. These results support further investigations to determine the role of dalbavancin in the treatment of BSI.

## Data Availability

My manuscript has associated data in a data repository, including all data for which depositation is mandatory.
